# 1326. Possible Predictors of Coinfection in COVID-19: Making a Difficult Diagnosis

**DOI:** 10.1093/ofid/ofab466.1518

**Published:** 2021-12-04

**Authors:** Christopher J Lehmann, Rohan N Shah, Mengqi Zhu, Natasha N Pettit, Jessica Ridgway, Renslow Sherer

**Affiliations:** 1 University of Chicago Medicine, Chicago, Illinois; 2 University of Chicago Pritzker School of Medicine, Chicago, Illinois; 3 University of Chicago Medicin, Chicago, Illinois

## Abstract

**Background:**

Coinfection with COVID-19 and a secondary pathogen contributes to morbidity and mortality. Despite its contribution to outcomes, diagnosing coinfection is challenging and no predictive tools have been established. To better assess risk factors for coinfection, we performed a review of all patients hospitalized for COVID-19 in our institution and evaluated them for candidate predictors of coinfection.

**Methods:**

Medical records were reviewed in all patients admitted with COVID-19 at University of Chicago Medical Center between March 1, 2020 and April 18, 2020. Those identified as having coinfection were compared to those without coinfection. Secondary review was performed for characteristics of the coinfection, including diagnosis, microbiology, drug resistance, and nosocomial acquisition.

**Results:**

401 patients were included in the study, the mean age was 60 years (SD-17), 29% had severe disease, and 13% died. At least one test for coinfection was performed in 99% of patients. Coinfection was identified in 15% (72/401) of patients. Coinfection was associated with older age, disease severity, and hospital complications, such as DVT/PE, AKI, and delirium. [Table 1] No symptom, non-microbiologic test, radiograph, or preexisting condition was associated with coinfection. Dyspnea, chest pain, and obesity were more common in those without coinfection. 74% received antibiotics. The most common sites for coinfection were urinary 33%, lower respiratory 26%, and blood 24%. [Table2] Bacteria were most frequently recovered (82%). The most commonly recovered pathogens were *Enterobacterales* (42%), *Staphylococcus aureus* (12%), and *Pseudomonas* (4%). 42% of the infections were hospital acquired, 16% caused by MDRO, and 13% were catheter or ventilator associated.

Table 1. Clinical Characteristics Associated with Coinfection

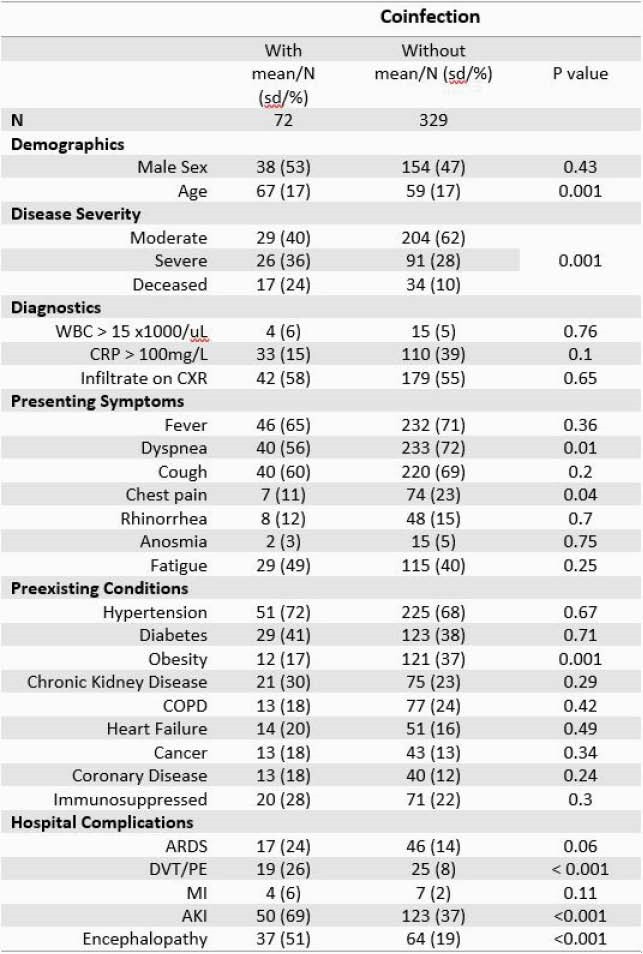

Abbreviations: sd, standard deviation; WBC, white blood cell count; CRP, C-reactive protein; COPD, chronic obstructive pulmonary disease; ARDS, acute respiratory distress syndrome; DVT, deep venous thrombosis; PE, pulmonary embolism; MI, myocardial infarction; AKI, acute kidney injury

Table 2. Characteristics of Coinfection

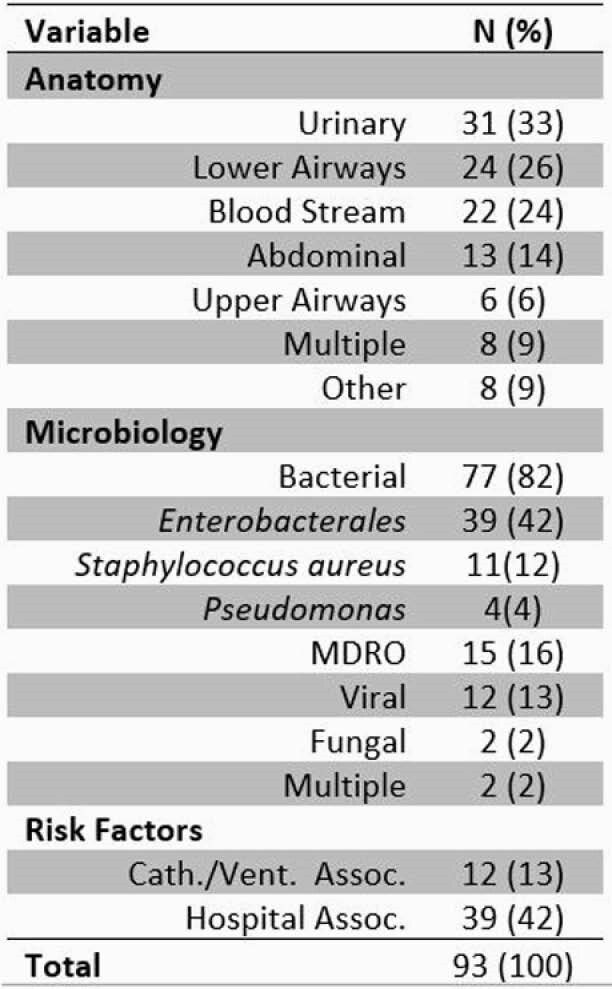

Abbreviations: Cath, catheter; Vent, ventilator; Assoc, Associated; MDRO, Multiple Drug Resistant Organism

**Conclusion:**

Coinfection in COVID-19 was most closely associated with age, COVID-19 disease severity, and complicated hospitalization. No presenting symptoms, non-microbiologic test, or radiograph was associated with coinfection, underscoring the challenge in diagnosing coinfection. A remarkable number of infections were hospital acquired, MDRO, and catheter/ventilator associated. Further prospective study on coinfection in COVID-19 is needed to guide diagnosis and treatment.

**Disclosures:**

**Renslow Sherer, MD**, **Gilead Sciences, Inc** (Grant/Research Support)

